# Stronger association of perceived health with socio-economic inequality during COVID-19 pandemic than pre-pandemic era

**DOI:** 10.1186/s12889-022-14176-8

**Published:** 2022-09-16

**Authors:** Je-Yeon Yun, Jin-Ah Sim, Sujee Lee, Young Ho Yun

**Affiliations:** 1 Seoul National University Hospital, Seoul, Republic of Korea; 2 Yeongeon Student Support Center, Seoul National University College of Medicine, Seoul, Republic of Korea; 3 Department of Family Medicine, Seoul National University Hospital, 101 Daehak-ro, Jongno-gu, Seoul, 03080 Republic of Korea; 4 School of AI Convergence, Hallym University, Chencheon, Republic of Korea; 5 Department of Industrial Engineering, Soongsil University, Seoul, Republic of Korea; 6 Department of Human System Medicine, Seoul National University College of Medicine, Seoul, Republic of Korea

**Keywords:** COVID-19, perceived health, socioeconomic position, Physical health, Mental health, Social health, Logistic regression model

## Abstract

**Objective:**

The COVID-19 pandemic has changed peoples’ routine of daily living and posed major risks to global health and economy. Few studies have examined differential impacts of economic factors on health during pandemic compared to pre-pandemic. We aimed to compare the strength of associations between perceived health and socioeconomic position (household income, educational attainment, and employment) estimated before and during the pandemic.

**Methods:**

Two waves of nationwide survey [on 2018(T1;*n* = 1200) and 2021(T2;*n* = 1000)] were done for 2200 community adults. A balanced distribution of confounders (demographics and socioeconomic position) were achieved across the T2 and T1 by use of the inverse probability of treatment weighting. Distributions of perceived health [= (excellent or very good)/(bad, fair, or good)] for physical-mental-social-spiritual subdomains were compared between T1 and T2. Odds of bad/fair/good health for demographics and socioeconomic position were obtained by univariate logistic regression. Adjusted odds (aOR) of bad/fair/good health in lower household income(< 3000 U.S. dollars/month) were retrieved using the multiple hierarchical logistic regression models of T1 and T2.

**Results:**

Perceived health of excellent/very good at T2 was higher than T1 for physical(T1 = 36.05%, T2 = 39.13%; *P* = 0.04), but were lower for mental(T1 = 38.71%, T2 = 35.17%; *P* = 0.01) and social(T1 = 42.48%, T2 = 35.17%; *P* < 0.001) subdomains. Odds of bad/fair/good health were significantly increased at T2 than T1 for household income (physical-mental-social; all Ps < 0.001) and educational attainment (social; *P* = 0.04) but not for employment (all Ps > 0.05). AORs of bad/fair/good health in lower household income were stronger in T2 than T1, for mental [aOR (95% CI) = 2.15(1.68–2.77) in T2, 1.33(1.06–1.68) in T1; aOR difference = 0.82(*P* < 0.001)], physical [aOR (95% CI) = 2.64(2.05–3.41) in T2, 1.50(1.18–1.90) in T1; aOR difference = 1.14(*P* < 0.001)] and social [aOR (95% CI) = 2.15(1.68–2.77) in T2, 1.33(1.06–1.68) in T1; aOR difference = 0.35(*P* = 0.049)] subdomains.

**Conclusions:**

Risks of perceived health worsening for mental and social subdomains in people with lower monthly household income or lower educational attainment became stronger during the COVID-19 pandemic compared to pre-pandemic era. In consideration of the prolonged pandemic as of mid-2022, policies aiming not only to sustain the monthly household income and compulsory education but also to actively enhance the perceived mental-social health status have to be executed and maintained**.**

## Introduction

The COVID-19 pandemic has been profoundly affecting our daily living, patterns of social network and communication, economic viability and healthcare functioning worldwide [1]**.** Higher risk of SARS-CoV-2 transmission has been associated with spending time in a bar, eating at a restaurant, and attending an indoor sporting event [2]. In this regard, changes of food-related behaviors such as lowered concern with choice of processed products and fast-food meals, as well as increased time use and efforts for home cooking have been reported [3]. With a fear of potential virus spread, both commuting and non-commuting travels have been reduced and avoidance of public transport such as airplanes and buses is consistently found across the countries during the COVID-19 pandemic [4]. Conversely, adherence to physical distancing is affected by anxiety and the prospect of economic losses by unemployment [5]. Researches of mental health in community population during the COVID-19 have reported moderate level of stress [6, 7], higher scores of depressive symptoms [6–9], anxiety [6–9], and symptoms of post-traumatic stress disorder [6]. Further, exposure of multiple waves of pandemic results in continual changes of risk perception, healthcare service use, and financial expenditure over time [10, 11]. At initial phase of pandemic, sense of isolation was suffered from half of the population with experience of lockdown [12]. In the later phase (late 2021), increased intensity of pain and fatigue were reported patients with pain disorder [13].

Not only before but also during the COVID-19 pandemic era [14], social determinants of health including the accessibility and quality of healthcare and education, social and community context, economic stability, neighborhood and built environment (https://www.cdc.gov) have important influence on health inequities (differences in the health status of individuals and groups). Regarding the physical health, vulnerable socioeconomic position such as higher income inequality, no enrollment in health insurance, housing overcrowding, and limited access to quality health care is associated with higher risk of COVID-19 mortality during the COVID-19 pandemic [14, 15]. Population density and socioeconomic inequality measured using the Gini index are correlated with a more rapid exponential growth in new cases and deaths [16, 17]. Moreover, socioeconomic disadvantage in a neighborhood’s mobility network has greater impact for subsequent incidence than its residents’ socioeconomic position [18]. In terms of the mental health during the COVID-19 pandemic, associations were found between the perceived mental health status versus physical health [12], mental factors of health overconfidence [19], self-compassion [20] and compassion from others [20], social factors of support from community [12, 21] and anticipated deterioration in social cohesion and security [22]. In addition, demographic factors of age [9], sex (male [6] or female [7, 9]), marital status (single [6, 7] or divorced [6]), familial size [7], type [6] and strength [12] of religious beliefs are associated with perceived mental health status. Further, socioeconomic position of current unemployment [8, 12], risks of unemployment [6, 7], lower monthly household income [6, 8, 12], and risk of reduced income [7] are related to poor mental health. Of note, degrees of perceived mental health and social health demonstrate positive correlation. For instance, suffer of a less strong social network and more loneliness, anxiety and depression are found in middle-aged people, people with a long-term health condition, and people receiving State financial benefits [23]. On the contrary, greater neighborhood identification is associated with a stronger social network and better mental health [23].

Recently, a few studies have examined the distributions of health status (health inequality) and socioeconomic position (socioeconomic inequality) as well as the strengths of association between these two phenomena during the COVID-19 pandemic. Inequality of perceived mental health seems to be increased during the peak periods of the COVID-19 pandemic [24]. On the contrary, the relative socioeconomic inequality did not increase [24] or became larger [25] during the COVID-19 pandemic era. Moreover, the magnitude of the impact of socioeconomic instability during the COVID-19 pandemic on perceived mental health varies with comorbid physical disease [26], demographics of age (> 45 years old [27] or < 26 years old [26]) and sex (women) [27], in addition to the socioeconomic position [27, 28] of lower household income [25, 27, 29–31], lower educational attainment [27, 29, 30], losing a job or becoming partially-employed [26, 30], housing disruptions [26] or renting housing [31]. For instance, exposure to the higher perceived risks of dying and of running out of money during pandemic have more detrimental effects on perceived mental health in people with poor socioeconomic position [23, 32, 33]. In short, study results regarding the distributions of socioeconomic position and perceived health in addition to the associations of perceived health status with socioeconomic position during the COVID-19 pandemic are scattered and diverse.

### Aim of the study

Till now, however, few studies applied multi-dimensional concept of perceived health in exploration of perceived health during the COVID-19. Further, although COVID-19 pandemic has changed peoples’ routine of daily living and posed major risks to global health and economy, little information is uncovered for possible changes of dynamic between the socioeconomic position versus perceived health during the pandemic compared to pre-pandemic era. Therefore, the current study compared distribution of perceived health (health inequality) in terms of the physical-mental-social-spiritual subdomains between the COVID-19 pandemic and pre-pandemic era. Also, effect sizes of demographics and socioeconomic position (household income, educational attainment, and employment) on perceived health during the COVID-19 pandemic were compared with those of pre-pandemic era. We hypothesized that inequalities of socioeconomic position and perceived health status would be larger during the COVID-19 pandemic than pre-pandemic era. Also, larger impacts of socioeconomic position of monthly household income for the perceived health status during the pandemic era compared to the pre-pandemic era were expected.

## Methods

### Study design and participants

The current study used dataset collected from two waves of nationwide survey conducted by way of the Computer Assisted Telephone Interviewing (CATI) in May 2018 (T1: pre-pandemic era) and between March and April 2021 (T2: during the COVID-19 pandemic). Target population was defined as follows: (1) adults aged 18 years or older, (2) currently living in Republic of Korea as of the year 2018 (for T1) or 2021 (for T2), (3) able to comprehend questionnaires written in Korean. By way of the probability-proportional-to-size sampling stratified for age and sex, target population that follows same distribution for age and sex with target population was sampled from panel database (*N* = 648,000) constructed by K stat (http://www.kstat.co.kr/). The contact information of participants used for the CATI in the current study had been obtained in the panel recruitment process of K stat (http://www.kstat.co.kr/). A total of 2200 community adults [in May 2018 (T1; *n* = 1200 participants among the candidates of *n* = 4000) and January 2021 (T2; *n* = 1000 participants among the *n* = 1800 candidates)], a subset of candidates who provided informed content, finally participated in the current study. This study was approved by the Ethics Committee of the Seoul National University Hospital (IRB No: 1804–024-934 for survey of T1 and IRB number: 2102–098-1197 for survey of T2) in compliance with the Declaration of Helsinki.

### Measurements

Perceived health status regarding the subdomains of physical, mental, social, spiritual, and general [34–37] were measured by 5 Health Status Questionnaire using the five-point Likert scale (excellent, very good, good, fair, or bad) [38, 39]. Firstly, physical health was defined as a state in which the body is not only free of diseases, wounds, etc., but also has normal physical strength. Second, mental health was described as a state being able to cope with stress and has a stable mood. Third, social health was explained as a state of maintaining social functioning and interpersonal relationship well. Fourth, spiritual health was defined as a state of having a clear reason for or meaning of life through volunteering, religious activities, and meditation, among others. Finally, participants were guided to score the general health considering the perceived state of physical, mental, social and spiritual health altogether. For statistical analyses, responses of bad/fair/good and ‘excellent/very good’ were binarized into the perceived health status of ‘poor’ and ‘ideal’, respectively [40].

In addition, information of demographics (age, sex, marital status, residential area, and religion) and socio-economic position (final education, monthly household income, and employment status) were gathered. For statistical analyses, responses of demographic were transformed into binary variables as follows: (1) sex [male (reference) vs. female], (2) age [< 65 years (reference) vs. ≥ 65 years], (3) marital status [married (reference) vs. unmarried/divorced/widowed], (4) residential area [urban (reference) vs. rural/suburban], (5) religion [having religion (reference) vs. not having religion]. Responses of socio-economic position were also converted into binary variables as follows: (1) final education [college graduation (reference) vs. ≤ high-school graduation], (2) employment status [employed/self-employed (reference) vs. unemployed/retired], (3) monthly household income [≥ 3000 U.S. dollars (reference) vs. < 3000 U.S. dollars (≈ 3rd quintile of monthly average income of households (with one or more family member) in Republic of Korea during 2019 and 2020 (https://kosis.kr/))].

### Statistical analysis

To obtain an unbiased average effect of exposure to the COVID-19 pandemic on inequality of perceived health, socioeconomic inequality, and odds of poor perceived health regarding the socioeconomic position, a balanced distribution of confounders (demographics and socioeconomic position) were achieved across the T2 and T1 by use of the inverse probability of treatment weighting (IPTW) [41–44]. All of the statistical analyses described below including the between-group comparisons and logistic regression analyses were performed using this confounder-balanced dataset. First, distributions of demographics and socioeconomic position (Table 1), in addition to the distributions of perceived health [= (excellent or very good)/(bad, fair, or good)] in five subdomains (Table 2), were compared between T1 and T2 using the Wald Chi-squared test [45]. Second, univariate logistic regression models of T1 and T2 were used to obtain the odds of bad/fair/good perceived health [odds ratio (OR) with a 95% confidence interval (95% CI)] regarding the demographics and socioeconomic position (Table 3). Third, using the hierarchical multiple logistic regression models of T1 and T2 (with entry and removal level of *P*-values< 0.05), odds of bad/fair/good perceived health for lower monthly household income (< 3000 U.S. dollars) compared to higher income, adjusted for demographics and employment status [adjusted odd ratio (aOR) with 95% CI] were also retrieved (Table 4). Finally, effect sizes of demographic or socioeconomic position for five subdomains of perceived health were compared between T2 versus T1 using the z-score normalization (http://genometoolbox.blogspot.com/2014/06/test-for-difference-in-two-odds-ratios.html). To test whether the two ORs are significantly different between T2 and T1, those of aforementioned z scores were calculated by the formula z = δ /SE(δ). Then, the *P* values of OR differences to identify two different time points (T2 and T1) were computed by way of the formula of *P* = 2 × (1-pnorm(z)). All calculated *P*-values were two-sided with the significance level set at *P* < 0.05. SAS statistical package version 9.3 (SAS Institute, Cary, NC, 1990) and R 3.5.1 were used for all analyses.

**Table 1:** Socio-demographic characteristics

		2018 (*N* = 1200)		2021 (*N* = 1000)		Wald F^c^	2018	2021	Walf F^c^ Adjusted for Propensity score^d^
		years	mean (SD)	years	mean (SD)	*p*-value			
Age (yrs)		46.97	14.18	47.96	14.66	0.27			
		N	%	N	%	*p*-value	%^a^	%^b^	*p*-value
Sex	Male	592	49.33	503	50.3	0.65	49.4	49.36	0.9786
	Female	608	50.67	497	49.7		50.6	50.64	
Age (yrs)	20–29	194	16.17	166	16.60	< 0.001	16.02	16.01	0.9781
	30–39	212	17.67	166	16.60		17.1	17.28	
	40–49	249	20.75	205	20.50		20.34	20.36	
	50–59	239	19.92	209	20.90		20.3	20.48	
	60–69	269	22.42	164	16.40		19.73	20	
	≥70	37	3.08	90	9.00		6.51	5.88	
Educational level	College graduate	539	44.92	541	54.10	< 0.001	49.27	49.26	0.9623
	Highschool graduate	537	44.75	361	36.10		40.54	40.79	
	Middle school or less	124	10.33	98	9.80		10.19	9.96	
Monthly household income	≥5000	249	20.75	276	27.60	< 0.001	24.19	23.84	0.9944
	4000-5000	300	25.00	275	27.50		26.05	26.11	
	3000-4000	344	28.67	228	22.80		25.99	26.09	
	< 3.000	307	25.58	221	22.10		23.77	23.96	
Marital Status	Not married	884	73.67	714	71.40	0.2	72.12	72.49	0.7856
	Married	316	26.33	286	28.60		27.88	27.51	
Residence	Urban	543	45.25	460	46.00	0.7	44.83	45.08	0.8659
	Rural/suburban	657	54.75	540	54.00		55.17	54.92	
Religion	Having religion	491	40.92	360	36.00	0.02	38.62	38.49	
	No religion	709	59.08	640	64.00		61.37	61.51	
Employment status	Occupied	840	70.00	747	74.70	0.01	72.2	62.2	0.9978
	Non-occupied	360	30.00	253	25.30		27.8	27.8	

^a^Percentage weighted to reflect all eligible participants. Before COVID group sample size = 1200, weighted = 2273.80

^b^After COVID group sample size = 1000, weighted = 2192.88

^c^F statistics based on Wald Chi-square test statistics

^d^Propensity score summarize difference in observable characteristics between before COVID group and after COVID group (i.e., age, sex, marital status, educational level, monthly household income, residential area, religion, and employment status etc.)

**Table 2:** Differential health status ‘during-‘versus ‘pre-‘COVID-19 pandemic, matched using the propensity score

		2018 (*N* = 1200)		2021 (*N* = 1000)		Wald F^c^	2018	2021	Walf F^c^ Adjusted For Propensity score^d^
		N	%	N	%	Chi-square (*p*-value)	%^a^	%^b^	Chi-square (*p*-value)
PHS	≥Very good	437	36.42	398	39.80	2.65 (0.10)	36.05	39.13	**4.44 (0.04)**
	<Very Good	763	63.58	602	60.20		63.95	60.87	
MHS	≥Very good	469	39.08	362	36.20	1.93 (0.16)	38.71	35.17	**5.93 (0.01)**
	<Very Good	731	60.92	638	63.80		61.29	64.83	
SHS	≥Very good	515	42.92	339	33.90	18.67 (< 0.001)	42.48	33.28	**39.58 (< 0.001)**
	<Very Good	685	57.08	661	66.10		57.52	66.72	
SpHS	≥Very good	338	32.33	301	30.10	1.26 (0.26)	31.78	29.31	3.17 (0.07)
	<Very Good	812	67.67	699	69.90		68.22	70.69	
GHS	≥Very good	421	35.08	305	30.50	5.18 (0.02)	34.42	30.04	**9.69 (0.002)**
	<Very Good	779	64.92	695	69.50		65.58	69.96	

^a^Percentage weighted to reflect all eligible participants. Before COVID group sample size = 1200, weighted = 2273.80

^b^After COVID group sample size = 1000, weighted = 2192.88

^c^F statistics based on Wald Chi-square test statistics

^d^Propensity score summarize difference in observable characteristics between before COVID group and after COVID group (i.e., age, income, education, etc.)

**Table 3:** Risk of poor perceived health status vs. demographic and socioeconomic position: ‘during-’ vs. ‘pre-’ COVID-19 pandemic

	Poor (<Very Good) PHS			Poor (<Very Good) MHS			Poor (<Very Good) SHS			Poor (<Very Good) SpHS			Poor (<Very Good) GHS		
	2018	2021	2018 vs. 2021	2018	2021	2018 vs. 2021	2018	2021	2018 vs. 2021	2018	2021	2018 vs. 2021	2018	2021	2018 vs. 2021
Variables	OR (95% CI)	OR (95% CI)	OR diff (*P*-value)	OR (95% CI)	OR (95% CI)	OR diff (*P*-value)	OR (95% CI)	OR (95% CI)	OR diff (*P*-value)	OR (95% CI)	OR (95% CI)	OR diff (*P*-value)	OR (95% CI)	OR (95% CI)	OR diff (*P*-value)
Sex															
Male	1 (Ref)	1 (Ref)		1 (Ref)	1 (Ref)		1 (Ref)	1 (Ref)		1 (Ref)	1 (Ref)		1 (Ref)	1 (Ref)	
Female	1.65 (1.38–1.96)	1.27 (1.07–1.51)	0.38† (0.03)	1.20 (1.02–1.43)	1.55 (1.30–1.85)	0.35† (0.01)	1.36 (1.15–1.61)	1.38 (1.15 1.65)	0.02 (0.87)	1.04 (0.87–1.24)	0.81 (0.67–0.97)	0.23 (0.08)	1.42 (1.19–1.69)	1.22 (1.02–1.47)	0.20 (0.12)
Age (years)															
< 65	1 (Ref)	1 (Ref)		1(Ref)	1 (Ref)		1 (Ref)	1 (Ref)		1(Ref)	1(Ref)		1 (Ref)	1 (Ref)	
≥ 65	6.32 (4.15–9.63)	4.16 (2.90–5.96)	2.16‡ (< 0.001)	4.77 (3.31–6.87)	2.35 (1.70–3.25)	2.42‡ (< 0.001)	3.44 (2.51–4.72)	4.28 (2.87–6.38)	0.84‡ (< 0.001)	4.62 (3.08–6.92)	2.10 (1.49–2.95)	2.52‡ (< 0.001)	5.28 (3.52–7.92)	4.26 2.81–6.53)	1.02‡ (< 0.001)
Education															
College graduate	1 (Ref)	1 (Ref)		1(Ref)	1 (Ref)		1(Ref)	1(Ref)		1(Ref)	1(Ref)		1(Ref)	1(Ref)	
≤ High-school graduate	2.65 (2.21–3.17)	2.55 (2.14–3.04)	0.1 (0.58)	2.17 (1.82–2.58)	2.12 (1.78–2.54)	0.05 (0.70)	2.33 (1.96–2.77)	2.59 (2.15–3.11)	0.26† (0.04)	2.17 (1.81–2.61)	1.83 (1.52–2.20)	0.34† (0.01)	2.46 (2.05–2.94)	2.33 (1.93–2.82)	0.13 (0.33)
Income															
≥ $3000	1 (Ref)	1 (Ref)		1(Ref)	1 (Ref)		1(Ref)	1(Ref)		1(Ref)	1(Ref)		1(Ref)	1(Ref)	
< $3.000	1.96 (1.57–2.45)	3.16 (2.50–3.99)	1.20‡ (< 0.001)	1.83 (1.48–2.27)	2.39 (1.90–3.01)	0.56‡ (< 0.001)	1.67 (1.36–2.05)	2.52 (1.99–3.20)	0.85‡ (< 0.001)	1.47 (1.18–1.83)	1.55 (1.23–1.95)	0.08 (0.62)	1.55 (1.25–1.93)	2.79 (2.16–3.60)	1.24‡ (< 0.001)
Marriage															
Married	1 (Ref)	1 (Ref)		1(Ref)	1 (Ref)		1(Ref)	1(Ref)		1(Ref)	1(Ref)		1(Ref)	1(Ref)	
Single	0.50 (0.42–0.61)	0.52 (0.43–0.63)	0.02 (0.88)	0.65 (0.54–0.79)	0.70 (0.58–0.85)	0.05 (0.72)	0.70 (0.58–0.84)	0.76 (0.63–0.93)	0.06 (0.66)	0.67 (0.55–.0.82)	0.96 (0.78–1.18)	0.29† (0.04)	0.49 (0.40–0.59)	0.71 (0.58–0.87)	0.22 (0.12)
Region															
Urban	1 (Ref)	1 (Ref)		1(Ref)	1 (Ref)		1(Ref)	1(Ref)		1(Ref)	1(Ref)		1(Ref)	1(Ref)	
Rural/ Suburban	0.93 (0.78–1.11)	0.85 (0.71–1.01)	0.08 (0.52)	0.99 (0.83–1.18)	1.08 (0.90–1.28)	0.09 (0.47)	1.01 (0.85–1.19)	1.00 (0.84–1.92)	0.01 (0.94)	0.84 (0.70–1.00)	1.65 (1.37–1.96)	0.81‡ (< 0.001)	0.89 (0.75–1.06)	0.92 (0.76–1.10)	0.03 (0.82)
Religion															
Having religion	1 (Ref)	1 (Ref)		1(Ref)	1 (Ref)		1(Ref)	1(Ref)		1(Ref)	1(Ref)		1(Ref)	1(Ref)	
No religion	0.66 (0.55–0.80)	0.71 (0.59–0.85)	0.05 (0.70)	0.78 (0.65–0.93)	0.74 (0.61–0.89)	0.04 (0.76)	0.84 (0.70–1.00)	0.76 (0.63–0.91)	0.08 (0.54)	1.05 (0.87–1.26)	0.90 (0.74–1.09)	0.15 (0.27)	0.92 (0.77–1.10)	0.78 (0.64–0.94)	0.14 (0.30)
Job status															
Occupied	1 (Ref)	1 (Ref)		1(Ref)	1 (Ref)		1(Ref)	1(Ref)		1(Ref)	1(Ref)		1(Ref)	1(Ref)	
Non-occupied	1.622 (1.33–1.99)	1.617 (1.33–1.97)	0.005(0.97)	1.51 (1.24–1.83)	1.59 (1.30–1.96)	0.08 (0.58)	1.71 (1.40–2.07)	1.66 (1.35–2.04)	0.05 (0.73)	1.40 (1.14–1.72)	1.24 (1.00–1.52)	0.16 (0.29)	1.76 (1.43–2.16)	1.44 (1.17–1.78)	0.32† (0.03)

*Abbreviation*: *PHS* Physical Health status, *MHS* Mental Health Status, *OR* Odds Ratio

†*P* < 0.05

‡*P* < 0.001

**Table 4:** Effect sizes for the level of monthly household income (socioeconomic inequality) on the level of perceived health status (health inequality) adjusted with other cofounders: ‘during-’ vs. ‘pre-’ COVID-19 pandemic

	Poor (<Very Good) PHS			Poor (<Very Good) MHS			Poor (<Very Good) SHS			Poor (<Very Good) SpHS			Poor (<Very Good) GHS		
	2018	2021	2018 vs. 2021	2018	2021	2018 vs. 2021	2018	2021	2018 vs. 2021	2018	2021	2018 vs. 2021	2018	2021	2018 vs. 2021
Variable	aOR^a^ (95% CI)	aOR^a^ (95% CI)	aOR diff (*P*-value)	aOR^a^ (95% CI)	aOR^a^ (95% CI)	aOR diff (*P*-value)	aOR^a^ (95% CI)	aOR^a^ (95% CI)	aOR diff (*P*-value)	aOR^a^ (95% CI)	aOR^a^ (95% CI)	aOR diff (*P*-value)	aOR^a^ (95% CI)	aOR^a^ (95% CI)	aOR diff (*P*-value)
Income															
≥ $3000	1 (Ref)	1 (Ref)		1 (Ref)	1 (Ref)		1 (Ref)	1 (Ref)					1 (Ref)	1 (Ref)	
< $3.000	1.50 (1.18–1.90)	2,64 (2.05–3.41)	1.14‡ (< 0.001)	1.33 (1.06–1.68)	2.15 (1.68–2.77)	0.82‡ (< 0.001)	1.33 (1.06–1.68)	2.15 (1.68–2.77)	0.35† (0.049)	–	–		1.33 (1.06–1.68)	2.15 (1.68–2.77)	1.05‡ (< 0.001)

*Abbreviation*: *PHS* Physical Health status, *MHS* Mental Health Status, *SHS* Social Health Status, *SpHS* Spiritual Health Status, *aOR* Adjusted Odds Ratio

^a^Adjusted with significant variables from the univariate analysis (age, sex, marriage, region, religion, job status) and weighted to reflect all eligible participants with propensity scores

†*P* < 0.05

‡*P* < 0.001

## Results

### Distribution of demographic and socioeconomic position: ‘during-’ vs. ‘pre-’ pandemic

Information of the study participants (*N* = 2200) regarding demographics and socioeconomic position are demonstrated in Table 1. Mean ± SD of age were 46.97 ± 14.18 years for T1 (*N* = 1, 200) and 47.96 ± 14.66 years for T2 (*N* = 1000), respectively. Percentage of male participants were 49.3% (T1) and 50.3% (T2). Percentage of college graduates (T2: 54.10% vs. T1: 44.92%) was higher in T2 than T1. Ratio of lower monthly household income (< 3000 U.S. dollars; T2: 54.25% vs. T1: 44.90%) and ratio of employed participants (T2 = 4.7% vs. T1 = 70.0%) were also higher in T2 compared to T1. However, socio-demographic differences were no longer significant in the confounder-balanced dataset after the propensity score analyses of IPTW.

### Distribution of perceived health status: ‘during-’ vs. ‘pre-’ COVID-19 pandemic

Percentages of poor perceived health (= bad/fair/good) were compared between the T1 and T2. All results are described with two versions of unweighted (‘Wald F’ column of Table 2) and weighted for the propensity score (‘Wald F adjusted for propensity score’ column of Table 2). Community adults on 2021 (T2) were more likely to report poor perceived health for subdomains of social (*P* < 0.001) and general (*P* = 0.02) than those on 2018 (T1). After the adjustment confounders were made using the IPTW, higher ratio of poor perceived health status was found for T2 in subdomain of mental [61.29% at T1, 64.83% at T2; Wald F adjusted for propensity score (adjusted F) = 5.93, *P* = 0.01], social (57.52% at T1, 66.72% at T2; adjusted F = 39.58, *P* < 0.001) and general (65.58% at T1, 69.96% at T2; adjusted F = 9.69, *P* = 0.002) compared to T1; on the contrary, ratio of poor perceived health status for physical subdomain was in T2 compared to T1 (63.95% at T1, 60.87% at T2; adjusted F = 4.44, *P* = 0.04).

### Risk of poor perceived health status vs. demographic and socioeconomic position: ‘during-’ vs. ‘pre-’ COVID-19 pandemic

The IPTW-weighed univariate logistic regression models (Table 3) indicated that odds of poor perceived health for high-school graduation (compared to the college graduation) at T2 was higher in social [OR (95% CI) = 2.33 (1.96–2.77) at T1 and 2.59 (2.15–3.11) at T2, OR difference = 0.26, *P* = 0.04] and was lower in spiritual [OR (95% CI) = 2.17 (1.81–2.61) at T1 and 1.83 (1.52–2.20) at T2, OR difference = 0.34, *P* = 0.01] subdomains compared to T1. Also, odds of poor general health for unemployed/retired status (compared to employed/self-employed status) was lower at T2 compared to T1 [OR (95% CI) = 1.76 (1.43–2.16) at T1 and 1.44 (1.17–1.78) at T2, OR difference = 0.32, *P* = 0.03]. Of note, odds of poor perceived health for lower monthly household income (< 3000 U.S. dollars; compared to ≥3000 U.S. dollars) were higher at T2 compared to T1 in subdomains of physical [OR (95% CI) = 1.96 (1.57–2.45) at T1 and 3.16 (2.50–3.99) at T2, OR difference = 1.20, *P* < 0.001], mental [OR (05% CI) = 1.83 (1.48–2.27) at T1 and 2.39 (1.90–3.01) at T2, OR difference = 0.56, *P* < 0.001], social [OR (95% CI) = 1.67 (1.36–2.05) at T1 and 2.52 (1.99–3.20) at T2, OR difference = 0.85, *P* < 0.001], and general [OR (95% CI) = 1.55 (1.25–1.93) at T1 and 2.79 (2.16–3.60) at T2, OR difference = 1.24, *P* < 0.001]. On the contrary, effect sizes of income level in the perceived spiritual health were comparable between T1 and T2 [OR (95% CI) = 1.47 (1.18–1.83) at T1 and 1.55 (1.23–1.95) at T2, OR difference = 0.08, *P* = 0.62].

For demographics, significant differences of effect size on poor perceived health between T1 and T2 were found in the physical (sex and age), mental (sex and age), social (age), spiritual (age, marriage, and residential area), and general (age) subdomains (all Ps < 0.05).

### Larger impacts of lower household income on perceived health status during pandemic than pre-pandemic

Table 4 shows effect sizes for the level of monthly household income (socioeconomic inequality) on the level of perceived health status (health inequality), adjusted for demographics and employment status (aOR) which were significant in the univariate analysis. In the IPTW-weighted hierarchical multiple logistic regression models, effects of lower monthly household income (less than 3000 US dollars) on the poor perceived health status were larger at T2 compared to T1 in subdomains of physical [aOR (95% CI) = 1.50 (1.18–1.90) at T1 and 2.64 (2.05–3.41) at T2, aOR difference = 1.14, *P* < 0.001], mental [aOR (95% CI) = 1.33 (1.06–1.68) at T1 and 2.15 (1.68–2.77) at T2, aOR difference = 0.82, *P* < 0.001], social [aOR (95% CI) = 1.33 (1.68–2.77) at T1 and 2.15 (1.68–2.77) at T2, aOR difference = 0.35, *P* = 0.049], and general [aOR (95% CI) = 1.33 (1.06–1.68) at T1 and 2/15 (1.68–2.77) at T2, aOR difference = 1.05, *P* < 0.001].

## Discussion

### Worse perceived mental and social health during the pandemic, compared to pre-pandemic era

The current study showed increased portion of bad/fair/good perceived health in mental and social subdomains during the COVID-19 pandemic than pre-pandemic era (Table 2). For perceived mental health, our result is in concordance with other studies that demonstrated higher perceived stress [46–48], depressive symptoms [49–51], anxiety [49–51], and burnout [50] during the pandemic than pre-pandemic era. Elderly population with lower global cognitive function is exposed to the higher odds of suffer from perceived stress and depressive symptoms during the COVID-19 pandemic [52]. Increased impact of older age on the level of perceived health during the COVID-19 pandemic could be mediated by changed patterns of social connectedness between the pre-pandemic versus during-pandemic era. Prosocial behavior, which is predicted by higher levels of perceived social support, is also related to the better well-being [51, 53]. Conversely, reduced mean number of social contacts compared to the pre-pandemic era has been maintained during the pandemic era from March 2020 to March 2021 [54]. Consequently, despite of the difficulties in activities of daily living (ADL) suffered from 18.4% of older adults living alone, inequality of providing ADL assistance during the pandemic has not been improved that much [55]. Perceived mental health is related to the perceived social health of loneliness [49, 51], perceived social support [46] and, organizational support [50], and home confinement [47].

In addition to the increased associations with monthly financial income and age, risk of poor perceived social health (in terms of social functioning and interpersonal relationship) in high-school graduation or lower educational attainment than college graduates was higher during the COVID-19 pandemic [OR (95% CI) = 2.59 (2.15–3.11)] than pre-pandemic era [OR (95% CI) = 2.33 (1.96–2.77)] (Table 3**).** Higher level of educational attainment facilitates social contact of elderly population (in their mid-60s) during the COVID-19 pandemic [56]. In addition to the availability of charitable assistance [57], educational attainment [13, 58] and monthly household income [13, 57–59] could affect treatment seeking intention, perceived physical health, and health-related quality of life in patients diagnosed with physical disease (such as pain disorder, stroke, or congenital heart disease). Moreover, college education could facilitate navigation and utilization of a complex healthcare system [60]. Conversely, poorer physical and mental functioning themselves could lead to the reduced intention of treatment seeking, worsening of perceived physical health, and lowered quality of life [59].

### Stronger associations of monthly household income with perceived health during pandemic

The current study results showed larger impact that lower monthly household income (socioeconomic inequality) has on perceived health status during the COVID-19 pandemic than pre-pandemic era. Poor socioeconomic position of lower household income itself has been associated with lower perceived mental health (mental well-being) [61]. On the contrary, better household wealth quantile is associated with less depressive symptoms by mediation of better healthcare service access and social contact [56]. Worse mental health of loneliness, anxiety, depression, and poor quality of life in people with lower financial status compared to better financial position both before and during the COVID-19 have already been reported [51]. Within communities of high deprivation, higher ratio of anxiety and acute behavioral disturbance is found from the male cases of mental health emergencies during COVID-19 pandemic (2020) than pre-pandemic era (2019) [62]. Parent-reported mental health problems are more likely to affect children with lower socioeconomic position [63]. Lack of compensatory source in the middle of unexpected financial crisis during the lockdown of COVID-19 pandemic in lower socioeconomic position could be associated with enlarged association of monthly income with perceived health in physical, mental, and social domains. Risk of developing distress financing is higher in households with poor economic position, with elderly family members, or with family members receiving inpatient care in the past 12 months [64]. Further, being in the lower socioeconomic position could be associated with a susceptibility to the influences from neighboring environmental factors. For instance, association between the availability of fast-food restaurants in the neighborhood versus obesity measured by body mass index is especially stronger in subgroup with lower monthly household income [65]. Also, residents of the most disadvantaged neighborhood have lower perceived mental health than those in the least disadvantaged neighborhoods, even after adjustment for individual-level socioeconomic position [61]. Individuals’ adaptive responses to the challenges of COVID-19 by applying the grit and resilience (among others) are paramount [66, 67]. Still, financial strain related to the lower financial income and unemployment could be associated with lesser interest in disease prevention (“not being ill”) and life expectancy (“living a long life”), respectively, of community adults [68].

### Strengths and limitations

The current study uncovered differential impact sizes of socioeconomic position features on perceived health status during the COVID-19 pandemic compared to pre-pandemic era. Since distribution of socioeconomic position was similar between the COVID-19 pandemic versus pre-pandemic era (Table 1), we could rule out the confounding effects of social mobility (= movement from one social subgroup to another) from the changes of perceived health status [69] between the COVID-19 pandemic versus pre-pandemic era. Moreover, by applying the multi-dimensional concept of perceived health, the current study could show distinctive patterns of socioeconomic position-by-pandemic interaction in each subdomains (physical-mental-social-spiritual) of perceived health. Conversely, the current study also has some limitations to be addressed. First, the current study did not gather follow-up assessment for the baseline (T1) participants; rather, between-group comparison with the separately recruited COVID-19 exposure group (T2) was done with covariate adjustment using the IPTW. Second, degree of association between self-perceived health versus objective health status such as life expectancy was not examined in the current study. Third, in regards of the prolonged exposure to the COVID-19 pandemic, additional data acquisition at T3 (during 2022) and comparison with the earlier period of pandemic (T2; January 2021) might be needed.

### Policy recommendations

Worsening of perceived mental health (in terms of stress coping and mood stability) and social health (regarding social functioning and interpersonal relationship) were found during the COVID-19 pandemic compared to pre-pandemic era. Especially those with lower household income, inclusion of screening for depressive symptoms, anxiety, and perceived stress using the self-reporting questionnaire within the program of national health checkup, combined with referral of supra-threshold cases to psychiatrists aiming to further evaluation and timely treatment, are required. Considering the increased influence of educational attainment and household income in perceived social health, public educational services for academic achievement of students and career restart of unemployed are in great need; responsive web-based educational program that applied recommendation system of artificial intelligence might be useful in providing a customized service during and after the pandemic. Further, policy research of how to maintain and recreate the social functioning and social connectedness of elderly after retirement has to be conducted.

## Conclusions

Changed daily routines and higher risks of health and economy during the COVID-19 pandemic are especially associated with more perceived hardships in maintaining the social functioning, interpersonal relationship, and stress coping, and with higher risks of mood instability and burnout, than pre-pandemic era. Of note, risks of perceived health worsening for mental and social subdomains in people with lower monthly household income and/or lower educational attainment became stronger during the COVID-19 pandemic compared to pre-pandemic era. In consideration of the prolonged pandemic as of mid-2022, policies aiming not only to sustain the monthly household income and compulsory education but also to actively enhance the perceived mental-social health status have to be executed and maintained.

## Data Availability

The datasets used and/or analyzed during the current study are available from the corresponding author (YHY) on reasonable request.
